# HERV-W association with serum biomarkers NfL and GFAP in multiple sclerosis

**DOI:** 10.3389/fimmu.2026.1776117

**Published:** 2026-03-23

**Authors:** Stefano Ruberto, Maria I. Dominguez-Mozo, Luisa María Villar, Lucienne Costa-Frossard, Noelia Villarrubia, Yolanda Aladro, Ignacio Casanova-Peño, Inés González-Suárez, María Angel Garcia-Martinez, Rafael Arroyo, Roberto Alvarez-Lafuente

**Affiliations:** 1 Grupo de Investigación de Factores Ambientales en Enfermedades Degenerativas, Instituto de Investigación Sanitaria del Hospital Clínico San Carlos (IdISSC), Red de Enfermedades Inflamatorias (REI), Red Española de Esclerosis Múltiple, Madrid, Spain; 2 Servicio de Inmunología, Hospital Universitario Ramón y Cajal, Red de Enfermedades Inflamatorias (REI), ISCIII, Red Española de Esclerosis Múltiple, Instituto Ramón y Cajal de Investigación Sanitaria, Madrid, Spain; 3 Servicio de Neurología, Hospital Universitario Ramón y Cajal, Red de Enfermedades Inflamatorias (REI), Red Española de Esclerosis Múltiple, Madrid, Spain; 4 Servicio de Neurología, Hospital Universitario de Getafe, Getafe, Spain; 5 Department of Neurology, University Hospital Torrejón, Torrejón de Ardoz, Getafe, Spain; 6 School of Medicine, Francisco de Vitoria University, Madrid, Spain; 7 Unidad de Enfermedades Desmielinizantes, Hospital Álvaro Cunqueiro, Red de Enfermedades Inflamatorias (REI), Vigo, Spain; 8 Departamento de Neurología, Hospital Universitario Quironsalud Madrid, Madrid, Spain

**Keywords:** biomarker, HERV-W, GFAP, NfL, multiple sclerosis

## Abstract

**Background:**

Serum biomarkers of multiple sclerosis (MS), such as glial fibrillary acidic protein (sGFAP) and neurofilament light chain (sNfL), are established indicators of disease progression and disability. Human endogenous retrovirus of the W family (HERV-W) is repeatedly associated with MS neuroinflammation, but its relationship with neural injury biomarkers is unclear.

**Methods:**

We measured anti-pHERV-W, -syncytin-1 IgG and their Ratio (HERV-W) in 83 RR-MS patients and 112 healthy controls (HC). Age and sex-adjusted sNfL/sGFAP Z-scores were derived from HC-calibrated GAMLSS models. HERV-W humoral responses were correlated with MS severity score (MSSS), cytokines (Olink™), and clinical phases (acute MS [AMS], stable MS [SMS]) *via* non-parametric tests and discriminant analysis.

**Results:**

HERV-W showed no difference between MS patients and HC, consistent with stable low-EDSS RR-MS predominance. HERV-W ratio correlated positively with Z-sNfL (ρ=0.67, p=0.012), Z-sGFAP (ρ=0.54, p=0.048), MSSS, and proinflammatory cytokines (IL-6, IL-1β and CXCL-9/10) specifically in AMS and SMS-EDSS>4 subgroups. AMS patients with elevated sNfL (>10 pg/ml; Z-score >1.5) exhibited markedly higher HERV-W than those with lower sNfl values. Discriminant models combining HERV-W, sNfL, and sGFAP achieved 82% of accuracy for MS-EDSS>4 classification; excluding HERV-W reduced the correct MS-EDSS>4 classification by 9.1%.

**Conclusions:**

HERV-W humoral activity shows state-specific associations with sNfL and sGFAP and proinflammatory status in acute and high-disability MS phases. These findings support integrating HERV-W humoral response into dynamic biomarker panels to better stratify patients by inflammatory burden and disability trajectory, positioning it as a cofactor linking immune dysregulation to neurodegeneration rather than a singular MS marker.

## Introduction

Multiple sclerosis (MS) is an immune-mediated disorder of the central nervous system (CNS) characterized by inflammation, demyelination, and neurodegeneration (1). Recent research has increasingly focused on fluid biomarkers that could help elucidate the immune-mediated mechanisms underlying disability and support early therapeutic decisions (2). Blood-based biomarkers are of particular interest due to their accessibility and minimal invasiveness.

Human endogenous retroviruses (HERVs), which constitute ~8% of the human genome, have emerged as potential biomarkers in MS (3). Among these, the HERV-W family has been consistently implicated in MS pathogenesis. Transcripts encoding envelope proteins (env) include Syncytin-1 (encoded by ERVWE-1) and a pathogenic protein called pHERV-W (encoded by MSRV) (3, 4). The latter may act as a superantigen *via* Toll-like receptor 4 (TLR4) (5), thereby exacerbating MS neuroinflammation (6). Concurrently, serum neurofilament light chain (sNfL) and glial fibrillary acidic protein (sGFAP) have been established as robust indicators of neural injury. Serum NfL reflects both clinical and radiological inflammatory activity and can forecast short- and long-term disability progression (7, 8). Serum GFAP, a marker of astrogliosis, is increasingly associated with progressive MS and disability regardless of inflammation, correlating with microstructural damage in normal-appearing white and gray matter (8, 9).

Combining HERV-W activity with sNfL and sGFAP could provide a more comprehensive understanding of MS pathology, bridging the gap between peripheral immune dysregulation and CNS tissue injury.

With growing interest in integrating immunological and neurodegeneration-related biomarkers, this study examines the humoral response to HERV-W and its association with inflammatory cytokines and direct biomarkers of neural injury, such as sNfL and sGFAP, in patients with distinct phases of MS and healthy controls. By combining these complementary biomarker areas, this study aims to provide new insights into disease progression mechanisms and assess the potential of HERVs as clinically relevant biomarkers in MS.

## Methods

### Data and sample collection

A total of 83 patients with MS and 110 healthy controls (HC) with comparable age and sex distribution were recruited from Hospital Clinico San Carlos in Madrid, according to McDonald criteria (10). MS participants were subdivided by sex, EDSS score (≤ 2; > 4), and disease phase (stable-MS, SMS: absence of disease activity in the 3 months before sample collection; acute-MS, AMS: relapses or MRI activity within two weeks of sample collection). Demographic and clinical data were collected from medical records or during study inclusion (**Supplementary Materials**). None of the participants received disease-modifying therapy (DMT) within the previous month. Additionally, no corticosteroids, intravenous immunoglobulins (IVIG), plasma exchange, or other immunomodulatory therapies were administered prior to sample collection. Peripheral blood was collected for PBMCs and serum isolation following standard procedure (11). The included participants provided informed consent via forms approved by the CEIm of the Hospital Clínico San Carlos (Approval Code: 17/177-E_BS). All procedures complied with the Declaration of Helsinki and local regulations.

### Serum and PBMCs clinical analysis

The humoral response to the HERV-W envelope protein family was evaluated using indirect ELISA for IgG detection, as described previously (12). The synthetic peptides tested were syncytin-1 486–500 (UniProt Q9UQF0) and pHERV-W 486–504 (UniProt Q991W9). Serum immunological factors were quantified with the Olink™ Target 48 Cytokine panel (Olink Signature Q100, 45 immune-related analytes, article no 932006, https://olink.com/products/olink-target-48). PBMCs were stimulated *in vitro* for intracellular cytokine staining, analyzed by flow cytometry (FACSDiva V.8.0, BD). IFN-γ, TNF-α, IL-17, and GM-CSF were assessed in CD4+ and CD8+ T cells, and TNF-α and GM-CSF in B cells. FACS analyses were performed as previously described by our group, according to the same gating strategy and compensation settings validated in that study (11). Serum levels of NfL and GFAP were quantified using the Simoa SR-X platform (Quanterix) (13).

### Statistical analysis

*Post-hoc* power analysis (G*Power 3.1.9.7) (14) confirmed 91.7% power (1-β=0.917) to detect medium effect sizes (Cohen’s d=0.5) between HC (n=112) and MS (n=83) using two-tailed Mann-Whitney U tests (α=0.05). Demographic variables (sex, age, age at onset) were compared between MS subgroups (EDSS ≤ 2 *vs* EDSS > 4; SMS *vs* AMS) using the Chi-square test for categorical variables and Student’s t test for continuous variables (**Supplementary Table 1**). Non-normally distributed data were expressed as median [IQR, 25th–75th percentile]. Non-parametric group comparisons were performed using the Kruskal–Wallis and Mann–Whitney U test. In the latter, the effect size was reported *via* Hodges–Lehmann median difference (Δ_HL_) and 95% CIs. Correlations were assessed using the Spearman rank test. Z-scores for sGFAP were computed from a GAMLSS model developed on the HC cohort, adjusting for age and sex. A Box–Cox t distribution was applied to generate age- and sex-specific reference curves, in analogy with reference models (15). Model parameters are reported in the **Supplementary Materials**. The same approach was applied to the age-adjusted sNfL values, enabling comparison with the Benkert et al. (2022) reference model (16) (**Supplementary Materials**). No covariate significantly affected the Ratio_HERV-W. Z-score thresholds were set according to prior studies (8, 17) (Z-sNfL > 1.5; Z-sGFAP > 1). Linear discriminant function analysis (LDFA) evaluated multivariate predictive models, validated by *leave-one-out* cross-validation. Analyses were performed with R v4.5.0, SPSS v28.0, and GraphPad Prism v8.0. Statistical significance was set at p ≤ 0.05.

## Results

### HERV-W humoral response and MS-related factors

**Figure 1:**
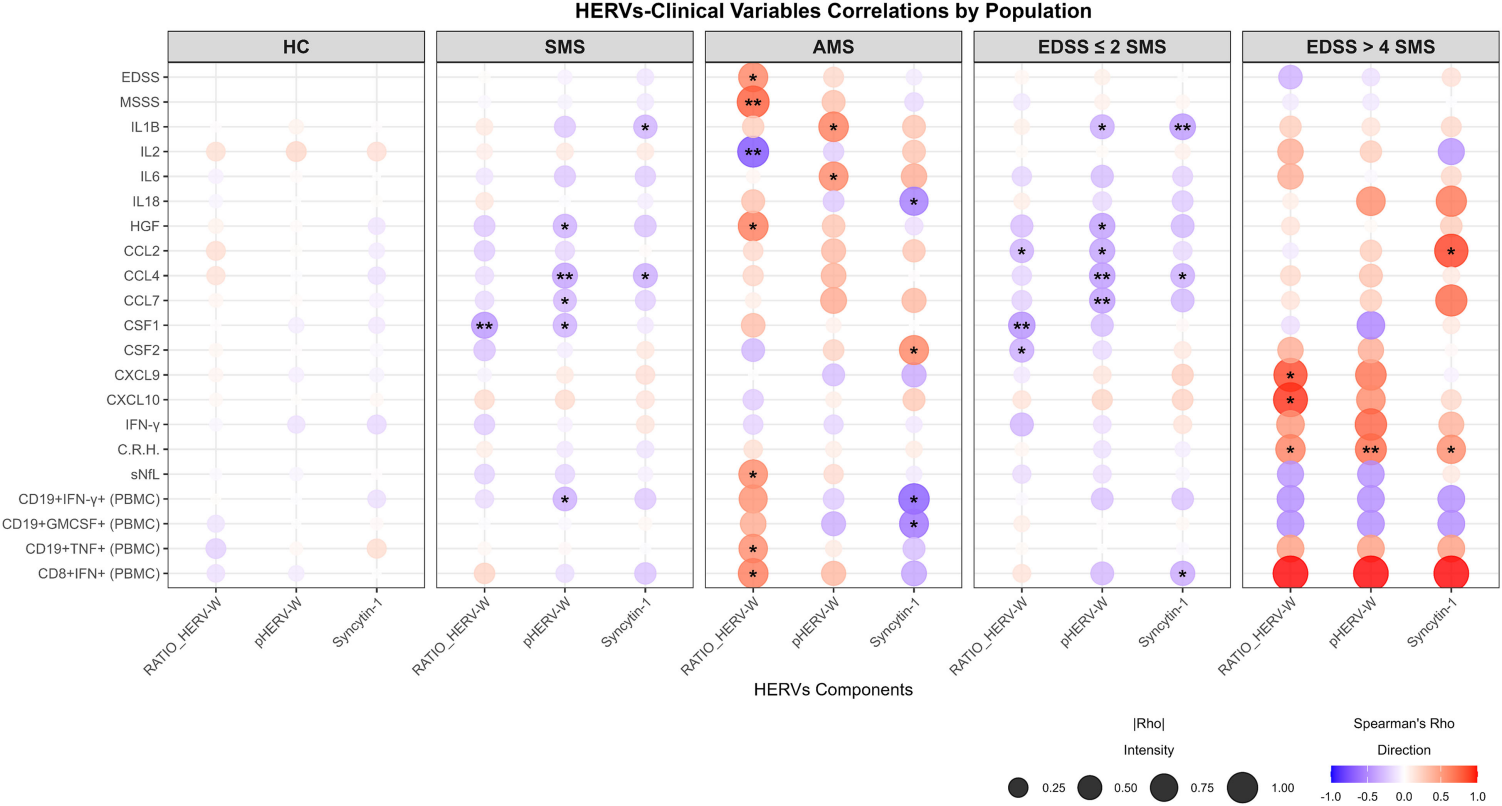
Bubble plot of Spearman’s correlation (r) between syncytin-1, pHERV-W, and Ratio_HERV-W in HC, the MS cohort, and MS subgroups *vs* MS-related variable and cytokine profiles: EDSS, Expanded Disability Status Scale; MSSS, Multiple Sclerosis Severity Score; IL1B, Interleukin-1 beta; IL2, Interleukin-2; IL6, Interleukin-6; IL18, Interleukin-18; HGF, Hepatocyte growth factor; CCL2, C-C motif chemokine 2; CCL4, C-C motif chemokine 4; CCL7, C-C motif chemokine 7; CSF1, Macrophage colony-stimulating factor 1; CSF2 Granulocyte-macrophage colony-stimulating factor; CXCL9, C-X-C motif chemokine 9; CXCL10, C-X-C motif chemokine 10; IFN-γ,Interferon gamma; C.R.H., Clinical Relapse History; sNfL, suero neurofilaments; CD19+INF-γ+ (PBMC), Interferon-γ in CD19+ PBMC-normalized; CD19+GMCSF+ (PBMC), GMCSF in CD19+ PBMC-normalized; CD19+TNF+ (PBMC), Tumor Necrosis Factor in CD19+ PBMC-normalized; CD8-+.IFN+ (PBMC), Interferon in CD8^+^ PBMC-normalized. *p ≤ 0.05, **p ≤ 0.01.

IgG antibody responses to syncytin-1env 486–500, pHERV-Wenv 486–504, and their ratio (pHERV-W/Synctyin-1; Ratio_HERV-W) did not differ significantly between HC and the overall MS cohort. Spearman correlations revealed several significant relationships between HERV-W elements, MS-related factors, and cytokines; no significant correlations were found in HC (**Figure 1**). For a more selective and accurate investigation, only associations with |*r*| ≥ 0.4 were considered. The strongest associations were observed in the AMS and SMS-EDSS >4 subgroup, where Ratio_HERV-W correlated strongly with MSSS (0.80), CCXL9 (0.89), and CCXL10 (0.94). These strong correlations for CXCL9/10 observed in the small SMS/EDSS > 4 subgroup warrant validation in larger cohorts.

### Ratio HERV-W and MS biomarkers

**Figure 2:**
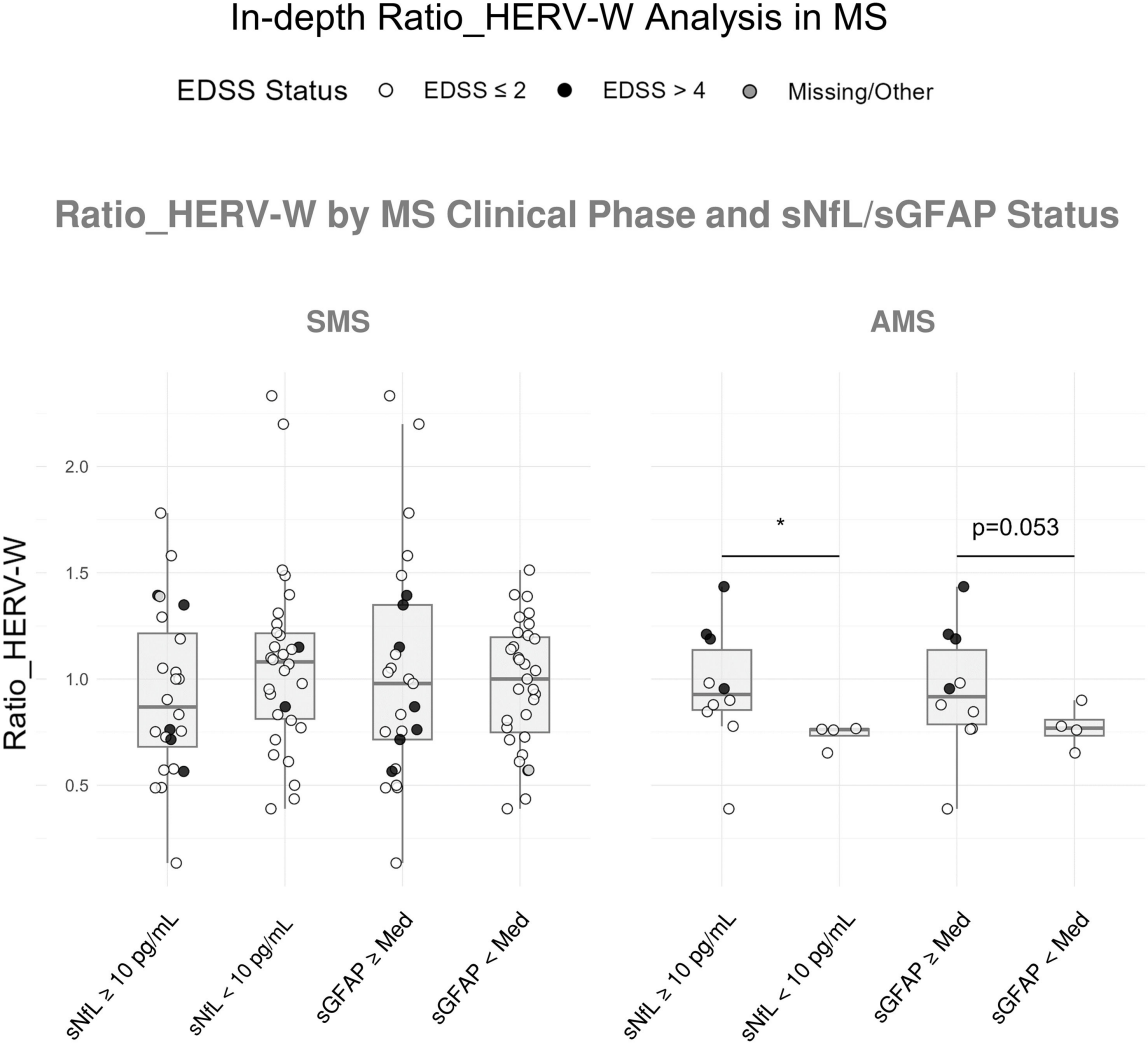
Association of Ratio_HERV-W with MS biomarkers profile. Ratio_HERV-W distribution according to NfL (below *vs*. above 10 pg/mL) and GFAP (below *vs*. above the median) levels in patients with Stable MS (SMS) and Acute MS (AMS). Dot colors according to the EDSS legend. Mann–Whitney tests; *p ≤ 0.05.

Ratio analysis suggested a higher, though nonsignificant, tendency in EDSS > 4 than ≤ 2 subgroups. Stratification by sNfL (cut-off: 10 pg/mL) and sGFAP (MS median cut-off), showed in AMS patients with sNfL > 10 pg/mL (median 0.93 [0.83-1.19]) significantly higher Ratio_HERV-W levels than those with sNfL ≤ 10 pg/mL (median 0.76 [0.68-0.77]; Δ_HL_ = 0.19, CI: 0.01/0.54, p = 0.024). A similar, though nonsignificant, trend was seen for sGFAP, with higher Ratio_HERV-W in the sGFAP-high subgroup. No difference was observed in the SMS group (**Figure 2**).

### Ratio HERV-W and MS biomarker Z score

Unlike sGFAP, whose Z-scores were derived from our internal reference model, Z-sNfL values were calculated using the Benkert et al. (2022) reference (16). The strong concordance between both models (Pearson’s r = 0.98; Bland–Altman bias = 0.37) supports the consistency of both approaches (**Supplementary Materials**). Therefore, given the larger sample size, the published reference model was adopted.

**Figure 3:**
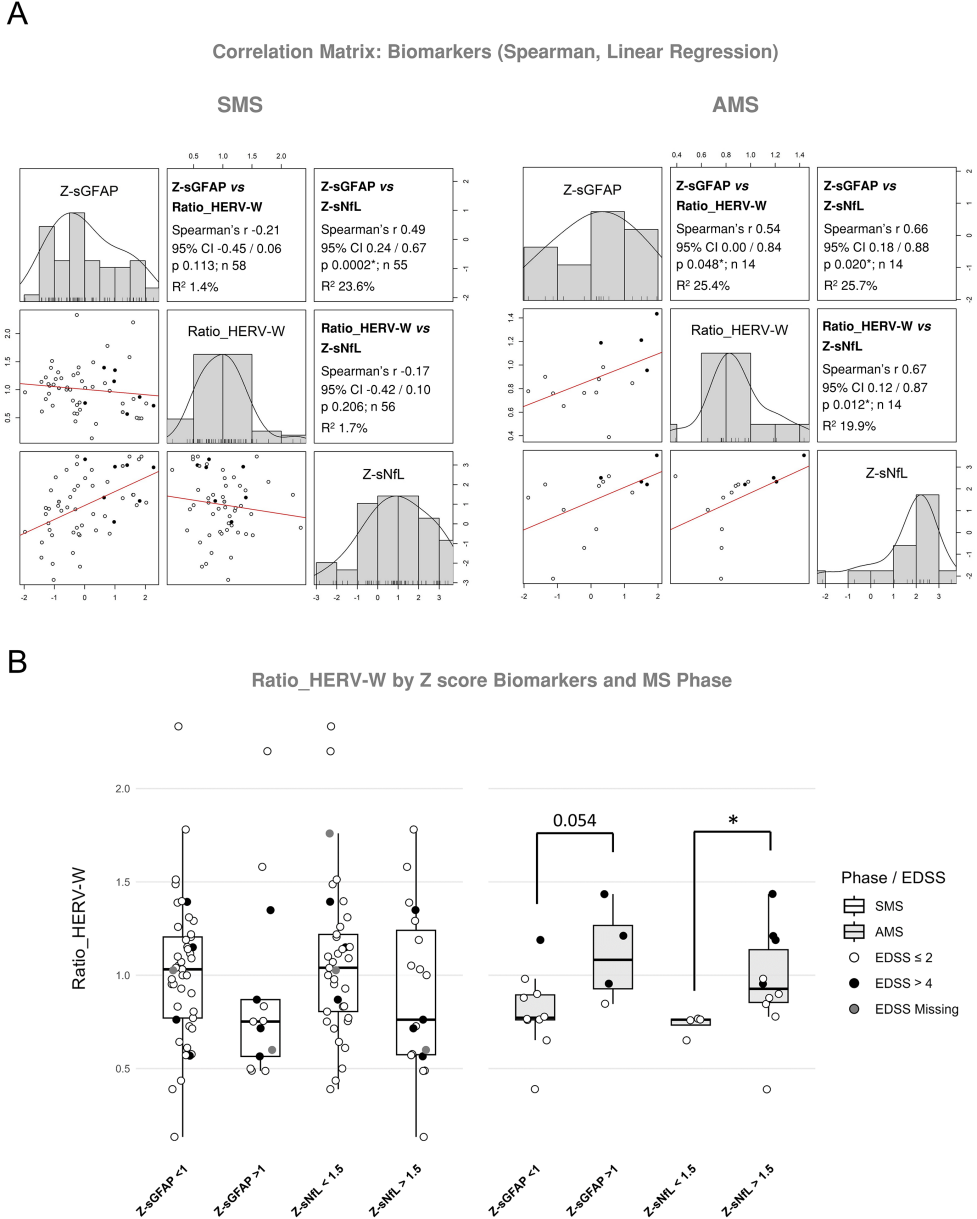
Association of Ratio_HERV-W with Z-score MS biomarkers by MS phase. **(A)** Matrix of correlation according to Spearman’s rho and linear regression analysis between Z-sGFAP, Ratio_HERV-W and Z-sNfL in Stable MS (SMS) and Acute MS (AMS). **(B)** Ratio_HERV-W distribution by Z-score cut-off for sNfL (1.5) and sGFAP (1) in SMS and AMS patients. *p ≤ 0.05.

Positive and strong correlations were identified between Ratio_HERV-W and both Z-sNfL and Z-sGFAP in AMS, but not in SMS (**Figure 3A**). In addition, Z-score cut-off analysis confirmed significantly higher Ratio_HERV-W levels in AMS with Z-sNfL > 1.5 (median 0.93 [0.83-1.19]) than Z-sNfL < 1.5 (median 0.76 [0.68-0.77]; Δ_HL_ = 0.19, CI: 0.01/0.54, p = 0.024), and a trend toward significance for Z-sGFAP (**Figure 3B**).

Finally, LDFA models demonstrated variability in discriminant accuracy according to the inclusion of Ratio_HERV-W. Excluding Ratio_HERV-W from the EDSS ≤ 2 *vs* > 4 model reduced classification accuracy by 6%. Across the combined HC, EDSS ≤ 2 and EDSS > 4 analysis, omitting HERV-W reduced correct classification by 9.1% within the EDSS > 4 cohort, underscoring its contribution to disease stratification (**Table 1**).

**Table 1:** Linear discriminant function analysis (LDFA) assessing the contribution of HERV-W–related humoral response to disability-based MS stratification.

Variance explained (1 - \Lambda)	Wilks’ \Lambda	*P*-value	Variable	Structure coefficient (DF)^§^	Classification accuracy (%)
EDSS ≤ 2 *vs* EDSS > 4					
24.9%	0.751	5.64e-10	Z-sGFAP	0.927	80.6
			Z-sNfL	0.644	
			Ratio_HERV-W	0.131	
*Excluding Ratio_HERV-W*					
24.0%	0.760	5.64e-10	Z-sGFAP	0.949	74.6
			Z-sNfL	0.659	
HC *vs* EDSS ≤ 2 *vs* EDSS > 4					
DF1					
33.8%	0.642	5.64e-10	Z-sGFAP	0.330	HC (70.3) EDSS **≤** 2 (42.9) EDSS > 4 (81.8)
			Z-sNfL	0.985^Ϯ^	
			Ratio_HERV-W	-1.13	
DF2					
9.3%	0.907	5.64e-10	Z-sGFAP	0.919^Ϯ^	Overall (61.9)
			Z-sNfL	-0.28	
			Ratio_HERV-W	0.263^Ϯ^	
*Excluding Ratio_HERV-W*					
DF1					
33.0%	0.670	5.64e-10	Z-sGFAP	0.346	HC (70.8) EDSS **≤** 2 (42.9) EDSS > 4 (72.7)
			Z-sNfL	0.994^Ϯ^	
DF2					
8.9%	0.911	5.64e-10	Z-sGFAP	0.938^Ϯ^	Overall (61.8)
			Z-sNfL	-0.105	

^§^Structure coefficients indicate the correlation between the variable and the latent discriminant function (DF). ^Ϯ^Largest absolute correlation between variables and function. Classification accuracy was estimated using *leave-one-out* cross-validation to account for unequal group sizes.

## Discussion

This study examined the role of HERV-W as a potential contribution alongside serum biomarkers reflecting peripheral inflammation and neural damage in MS. Several studies have shown that HERV-W/MSRV activity in the MS cohort was not uniform across patients and appeared to be influenced by disease activity and evolution, disability burden, and biomarker-defined profiles (18–20), especially in the biological fluids of MS patients with active disease (21). These studies support that the lack of a significant difference between MS patients and HC is likely attributable to the clinical composition of the cohort, which consisted predominantly of RR-MS patients in a stable phase (~80%) with limited inflammatory activity. Accordingly, the Ratio_HERV-W was not intended as a diagnostic discriminator between MS and HC but rather as a relative index of humoral reactivity, enabling the exploration of intra-cohort biological associations with inflammatory and neurodegenerative biomarkers, and acts as a state-dependent immunological contextual marker rather than an intrinsic predictive biomarker. Further stratified analyses were limited by the unbalanced distribution of clinical subgroups, with relatively few patients in the acute phase and a small number of RR-MS patients with higher disability scores (EDSS > 4) compared to HC. Despite these limitations, the observed pattern is consistent with previous reports suggesting that HERV-W activity, and particularly its humoral response, is more closely associated with progressive or disability-related disease states rather than with multiple sclerosis *per se*. Along these lines, HERV-W levels were also associated with clinical and inflammatory markers, such as EDSS, sNfL, and several proinflammatory cytokines, particularly in AMS and SMS patients with EDSS >4, supporting its contribution to disease progression and disability (19, 22).

In acute-phase patients with elevated sNfL, the enhanced anti-HERV-W response suggests that HERV-W may act as a damage-associated molecular pattern (DAMP), amplifying inflammatory cascades. The strong correlations observed between HERV-W and Z-score MS-biomarkers in AMS could be mediated by TLR4-dependent pathways, consistent with previous reports showing microglial activation through TLR4 in CNS neurodegeneration (23–25). The established link between HERV-W, TLR4 activation (5), and subsequent nuclear factor-κB (26) signaling supports its role as a cofactor in chronic glial inflammation.

Furthermore, the strong correlation between pro-inflammatory mediators (IFN, IL-6, IL-1β) and chemoattractants (CXCL9/10) exclusively in AMS and SMS (with EDSS>4) cohorts reinforces the relationship between HERV-W activity and astrogliosis (27, 28), which may drive acute inflammatory processes reflected by increased sNfL levels (29).

Finally, the Ratio_HERV-W factor significantly impacted discrimination between MS subgroups, highlighting its potential predictive value and supporting its interaction with both sNfL and sGFAP, particularly within the MS cohort with EDSS > 4. These results reinforce the concept that HERV-W–related immune responses are not uniformly associated with MS *per se*, but rather with specific disease states characterized by increased disability burden or biomarker-defined neural injury, supporting its role as a contextual marker integrated with sNfL and sGFAP.

These findings should be interpreted cautiously due to the small sample size of individual subgroups, which may limit statistical power and render these analyses exploratory and hypothesis-generating, rather than definitive or generalizable to the overall MS population. Additionally, recruiting individuals in the acute phase with low sNfL and sGFAP levels is inherently difficult. This may hinder the assessment of biomarker interactions in acute MS cases with minimal neuroaxonal injury.

Nevertheless, the consistent associations of HERV-W across multiple clinical profiles strengthen the biological validity of the findings. Moreover, using a model calibrated from the same HC cohort enhances the internal consistency of the calculated Z-scores.

These data add to emerging evidence for an integrated relationship between HERV-W, sNfL, and sGFAP, reflecting distinct but complementary mechanisms of MS pathophysiology, including peripheral inflammation, astroglial activation, and axonal injury. Larger longitudinal studies are essential for defining the specific contribution of endogenous retroviruses to the different stages of the disease. In addition, studies correlating HERV-W responses with radiological findings and neuromyelitis optica spectrum disorders are warranted to validate and extend these observations.

Combined with established MS biomarkers, this could help to clinically stratify patients, identify those at higher risk of progression, and improve therapeutic decision-making in the early stages of the disease.

Overall, these findings suggest that HERV-W activation may reflect neuroinflammation, supporting glial activation and axonal damage and linking immune dysregulation and neurodegeneration in MS. These observations position HERV-W as a potentially useful immune-neurodegeneration link and contextual biomarker, suggesting that HERV-W activity, and particularly its humoral response, is more closely associated with progressive and/or disability-related disease states rather than with MS *per se.*

## Data Availability

The raw data supporting the conclusions of this article will be made available by the authors, without undue reservation.
