# Supplementary material for: HERV-W association with serum biomarkers NfL and GFAP in multiple sclerosis
Source: Front Immunol. 2026 Mar 23;17:1776117. doi: 10.3389/fimmu.2026.1776117 (PMC13050694; doi:10.3389/fimmu.2026.1776117)
Supplement: Supplementary file 1 [file DataSheet1.docx]

**Supplementary Materials**

**s. Table 1.**

Clinical and demographic characteristics of the study population.

**s. Methods 1.**

Statistical Analysis

**s.Table 2.**

Estimated Coefficients of the Box-Cox t (BCT) GAMLSS Model for log (GFAP+1) in the Healthy Control Cohort.

**s.Table 3.**

Estimated Coefficients of the Box-Cox t (BCT) GAMLSS Model for log (NfL+1) in the Healthy Control Cohort.

**s.Figure 1.**

Quantile-Quantile (QQ) Plots of Standardized Z-scores (HC)

**s.Figure 2.**

Diagnostic Plot for Standardized Z-scores (Z-sGFAP) vs Age (HC) (A). Diagnostic Box Plot for Sex Effect Removal (HC) (B).

**s.Figure 3.**

Diagnostic Plot for Standardized Z-scores (Z_NfL) vs Age (HC).

**s.Figure 4.**

Age-related sGFAP (pg/mL) Reference Centile Curves in Healthy Controls (HC)

**s.Figure 5.**

Age-related sNfL (pg/mL) Reference Centile Curves in Healthy Controls (HC)

**s.Figure 6.**

External Validation and Agreement Analysis of Locally Derived NfL Z-scores vs Standardized Reference in HC and MS.

**s.Table 1.** Clinical and demographic characteristics of the study population.

| Variables | HC | Total MS Population^§§^ | MS Patient Subgroups | | | | | |
| --- | --- | --- | --- | --- | --- | --- | --- | --- |
|  |  |  | **Female** | **Male** | **EDSS ≤ 2** | **EDSS > 4** | **SMS** ^Ϯ^ | **AMS** ^Ϯ Ϯ^ |
| n | 112 | 83 | 60 | 23 | 59 | 21 | 67 | 16 |
| Age, years  (Mean ±SD) | 39 ± 9.4 | 38 ± 8.8 | 38 ± 8.7 | 39 ± 9.2 | 38 ± 8.3 | 40 ± 10.3 | 38 ± 8.9 | 38 ± 8.7 |
| Sex, n  (Male/Female) | 26/86 | 23/60 | - | - | 13/46 | 9/12 | 19/48 | 4/12 |
| EDSS^§^  [Median (Range)] | - | 1 (0-7) | 1 (0-7) | 1 (1-5) | - | - | 1 (0-7) | 1 (0-5) |
| Age at onset, years (Mean ±SD) | - | 30 ± 8.4 | 30 ± 7.6 | 30 ± 10.3 | 31 ± 8 | 27 ± 9 | 30 ± 8.5 | 29 ± 8.1 |

^§^Expanded Disability Status Scale; ^Ϯ^ stable-MS, absence of disease activity in the 3 months before sample collection; ^ϮϮ^ acute-MS, relapses and/or evidence of MRI activity within two weeks of symptoms onset. No statistical differences were observed between EDSS ≤ 2 and EDSS > 4 or between SMS and AMS in terms of sex (Chi-square test) and age/age at onset (Student’s t test). ^§§^ All MS samples were collected ≥1 month after DMT discontinuation. Therapy history: naïve 51% (n=42), moderate-efficiency treatments (MET) 36% (n=30), high-efficiency treatments (HET) 13% (n=11; Natalizumab n=5, Alemtuzumab n=2, Azathioprine n=2, Fingolimod n=2). No anti-CD20 therapies administered.

**s. Methods 1.**

**Statistical Analysis**

The relationship between serum NfL (sNfL) and serum GFAP (sGFAP) levels and physiological variables was analyzed exclusively in the Healthy Control (HC) cohort to establish age-specific normative curves. Concentrations of both biomarkers were transformed using the log (biomarker + 1) function to correct for marked right skewness and accommodate zero values. The HC reference cohort comprised 110 individuals spanning the adult age range (18–60 years). All MS participants were within the modeled range, ensuring that Z-score estimation was based on interpolation within the established HC reference model.

A Generalized Additive Model for Location, Scale, and Shape (GAMLSS), based on a Box-Cox t (BCT) distribution, was employed to model the non-linear age dependency and variance structure for each biomarker. The final parsimonious model for each biomarker was selected according to the Akaike Information Criterion (AIC) and confirmed by residual diagnostics QQ Plots (s. Fig.1).

Final Model Specifications:

- sGFAP Model: Included Age (modeled as a pb-spline in the location parameter, μ) and Sex (included as a linear predictor) to account for physiological variation (s. Fig. 2).
- sNfL Model: Included Age (modeled as a pb-spline in the parameter μ), resulting in a more parsimonious model (s. Fig. 3).

Z-scores were then calculated for each subject using the estimated parameters from the GAMLSS model, effectively removing the physiological effect of age (and sex, for GFAP). To ensure maximum generalizability and clinical consistency, the locally derived Z-scores were cross-validated against Z-scores obtained from a standardized external reference model (16). Bland-Altman agreement analysis and Pearson correlation (r = 0.98) confirmed an excellent agreement between the two methods. Based on this concordance and the larger sample size of the reference cohort, the published reference model was adopted for subsequent analyses. P-value below 0.05 were considered statistically significant. Analyses were performed in R v4.5.0.

| GAMLSS Regression Model Results for log (GFAP+1) in Healthy Controls | | | | | | |
| --- | --- | --- | --- | --- | --- | --- |
| Term | **Estimate** | **Std. Error** | **t value** | **95% CI Lower** | **95% CI Upper** | **P-value** |
| μ (Location) | | | | | | |
| Intercept | 3.892 | 0.157 | 24.810 | 3.585 | 4.200 | < 0.001 |
| Sex [Male] | −0.252 | 0.072 | −3.501 | −0.393 | −0.111 | **< 0.001** |
| pb(Age)  *(Non-Linear Smooth)* |  |  |  |  |  | **0.037** |
| σ (Scale) | | | | | | |
| Intercept | −1.804 | 0.316 | −5.712 | −2.424 | −1.185 | < 0.001 |
| pb(Age)  *(Non-Linear Smooth)* |  |  |  |  |  | **0.019** |
| ν (Skewness) | | | | | | |
| Intercept | 0.930 | 0.933 | 0.997 | −0.898 | 2.758 | 0.321 |
| τ (Kurtosis) | | | | | | |
| Intercept | 4.280 | 5.457 | 0.784 | −6.416 | 14.977 | 0.435 |

**s.Table 2.** Estimated Coefficients of the Box-Cox t (BCT) GAMLSS Model for log (GFAP+1) in the Healthy Control Cohort

**Model and Distribution:** Results are derived from a Generalized Additive Model for Location, Scale, and Shape (GAMLSS) using the Box-Cox t (BCT) distribution for log (GFAP+1) in the Healthy Control (HC) group (N=110).

**pb (Age):** penalized B-Spline smoothing term, indicating a non-linear relationship with Age. The P-value listed assesses the overall significance of this non-linear effect. Numerical estimates and standard errors are omitted for smooth terms as they do not represent a single, linear coefficient.

**µ (Location):** Sex [Male] shows a significant negative association (Est. -0.252, P < 0.001), meaning log (GFAP+1) levels are, on average, lower in HC males compared to HC females (the reference group).

**σ (Scale):** The significant pb (Age) effect (P=0.019) suggests that the variability of log (GFAP+1) is not constant with Age.

**s.Table 3.** Estimated Coefficients of the Box-Cox t (BCT) GAMLSS Model for log (NfL+1) in the Healthy Control Cohort

| GAMLSS Regression Model Results for log (NfL+1) in Healthy Controls | | | | | | |
| --- | --- | --- | --- | --- | --- | --- |
| Term | **Estimate** | **Std. Error** | **t value** | **95% CI Lower** | **95% CI Upper** | **P-value** |
| μ (Location) | | | | | | |
| Intercept | 1.160 | 0.120 | 9.629 | 0.925 | 1.395 | < 0.001 |
| pb(Age)  *(Non-Linear Smooth)* | 0.019 | 0.003 | 5.954 | 0.013 | 0.025 | **< 0.001** |
| σ (Scale) | | | | | | |
| Intercept | −1.941 | 0.129 | −14.97 | -2.194 | -1.688 | < 0.001 |
| ν (Skewness) | | | | | | |
| Intercept | -0.382 | 0.621 | -0.616 | -1.599 | 0.835 | 0.540 |
| τ (Kurtosis) | | | | | | |
| Intercept | 2.208 | 1.005 | 2.197 | 0.238 | 4.178 | **0.030** |

**Model and Distribution:** Results are derived from a Generalized Additive Model for Location, Scale, and Shape (GAMLSS) using the Box-Cox t (BCT) distribution for log (NfL+1) in the Healthy Control (HC) group (N=110).

**pb (Age):** penalized B-Spline smoothing term, indicating a non-linear relationship with Age. The P-value listed assesses the overall significance of this non-linear effect. The smoothing term was treated as a penalized linear component within the model's design matrix.

**τ (Kurtosis):** The parameter modeling the heaviness of the tails (kurtosis) of the distribution. It allows the model to better fit data with more extreme outliers than would be assumed by a normal distribution. τ was included as a constant intercept.

**s.Figure 1.** Quantile-Quantile (QQ) Plots of Standardized Z-scores (HC)


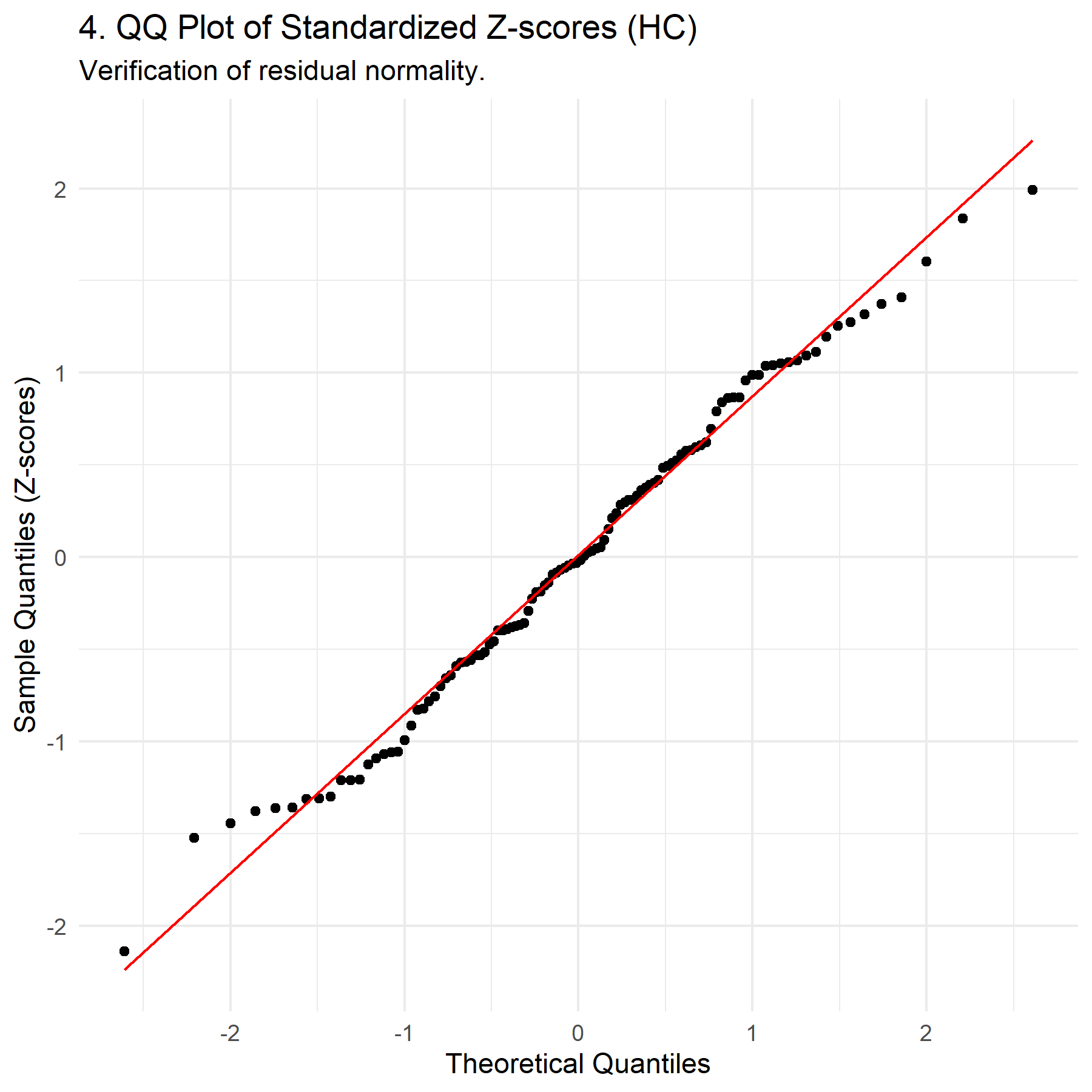

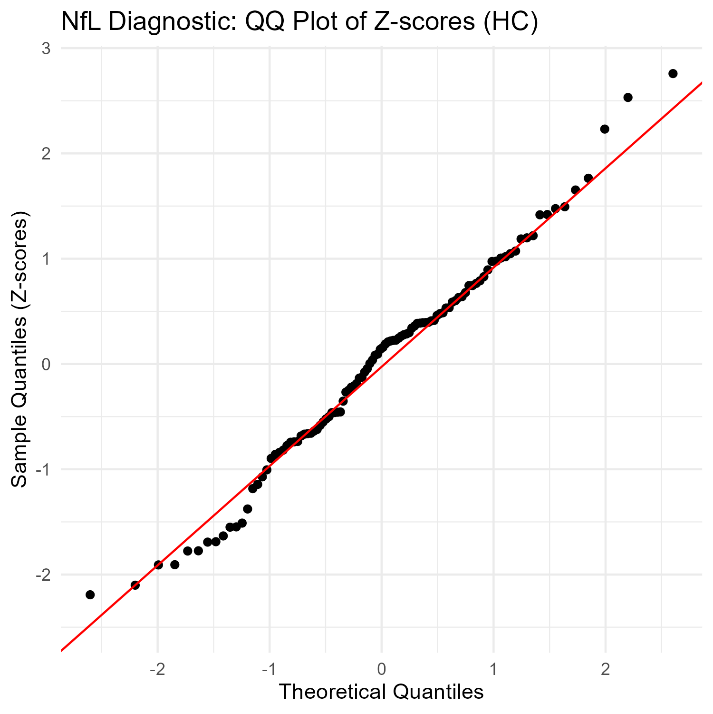

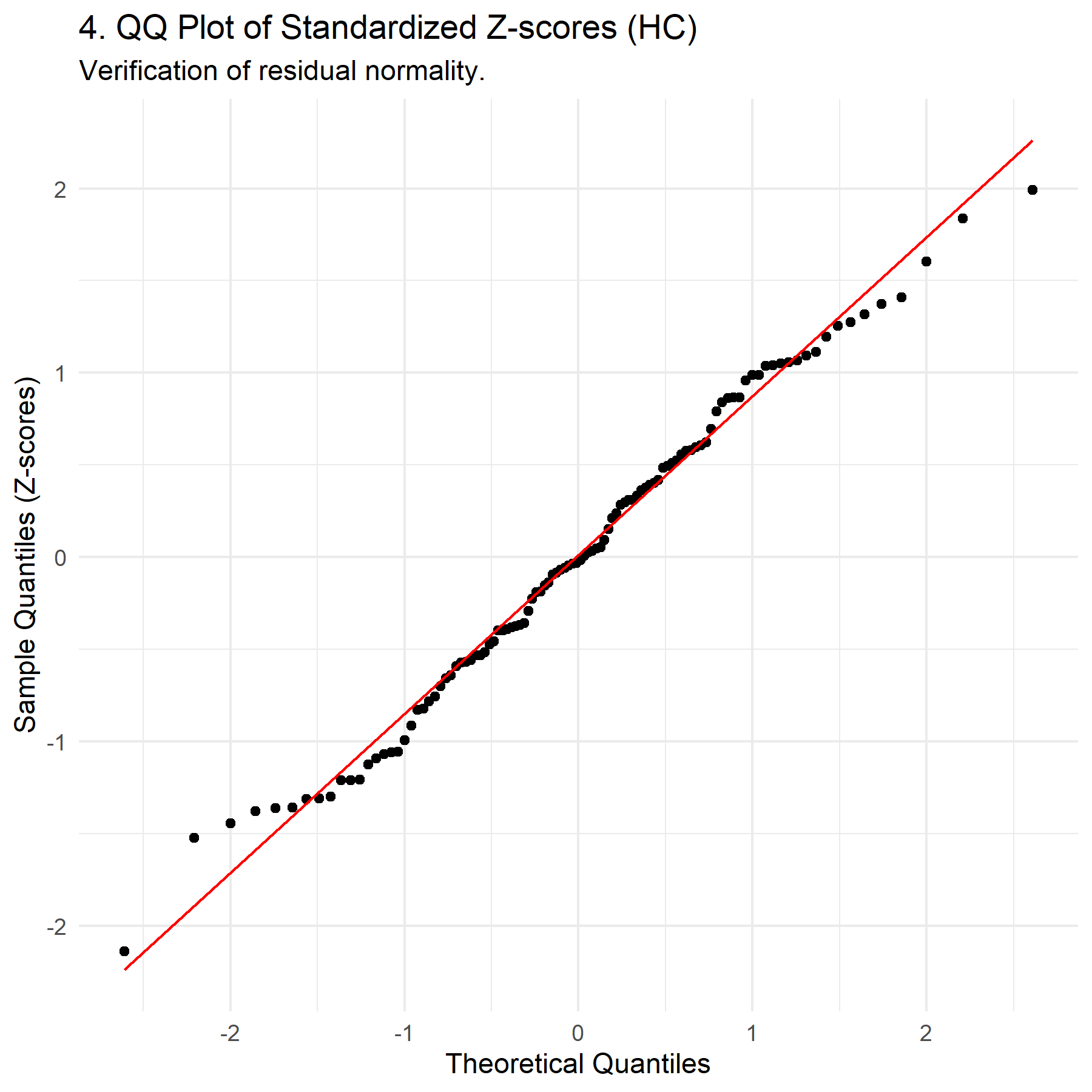

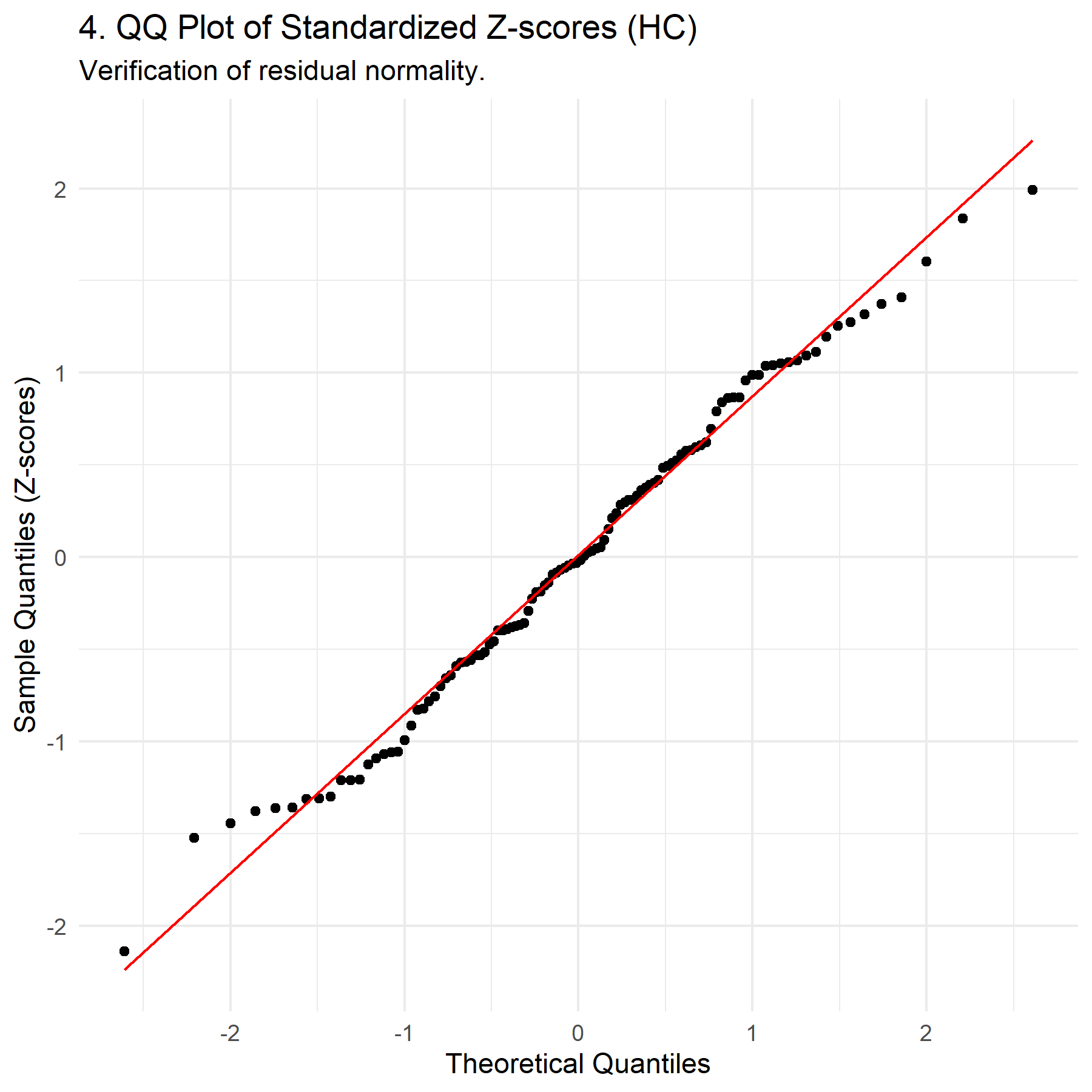


**GFAP NfL**

Legend: The QQ Plot was used to assess the distributional assumption of the residuals. The data quantiles (Y-axis) plot closely against the theoretical standard normal quantiles (X-axis) along the red diagonal line. The distribution confirms that the Box-Cox t transformation and the subsequent standardization process successfully transformed the original GFAP and NfL data into normally distributed residuals.

**s.Figure2.** Diagnostic Plot for Standardized Z-scores (Z-sGFAP) vs Age (HC) (A). Diagnostic Box Plot for Sex Effect Removal (HC) (B)

**
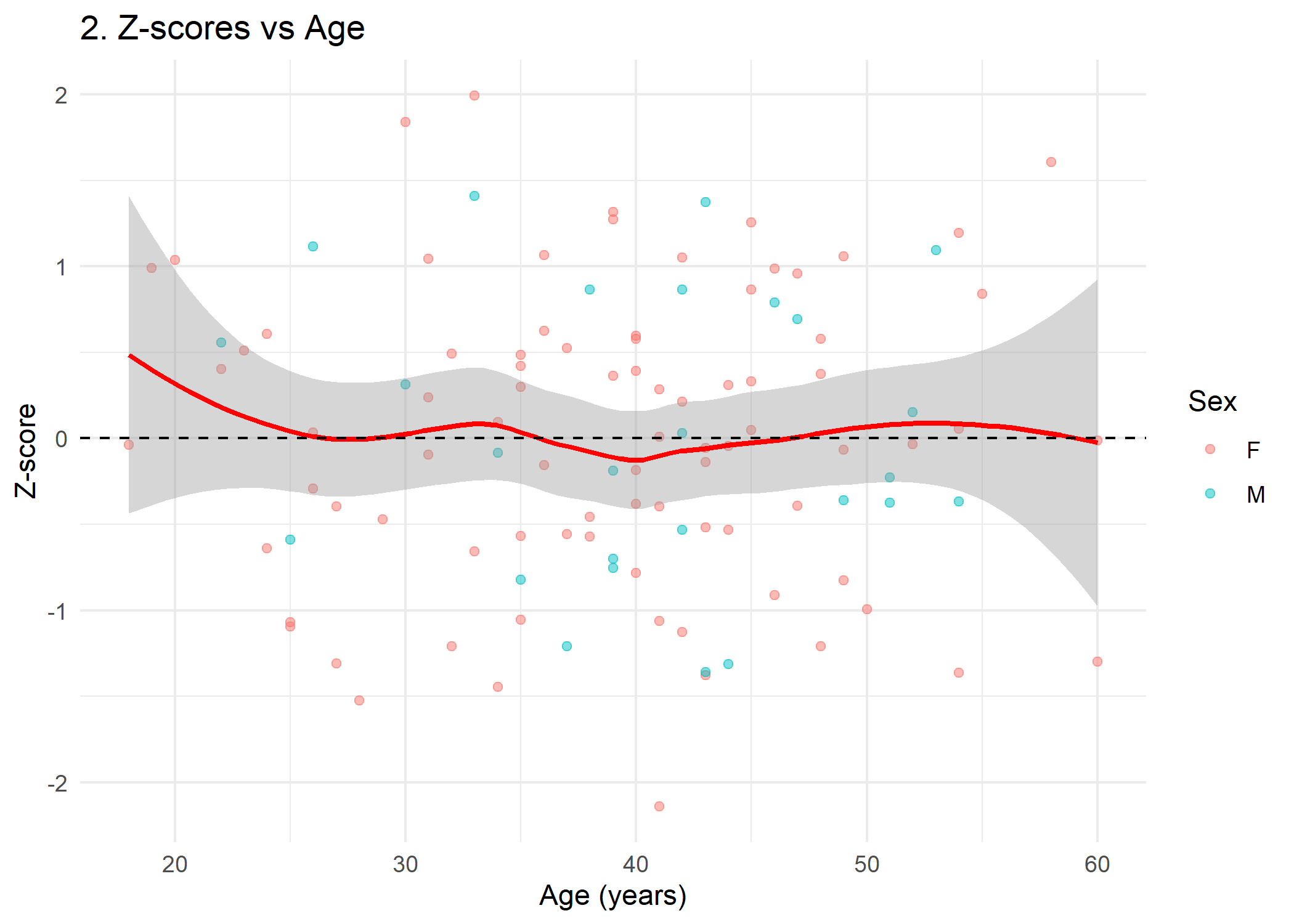

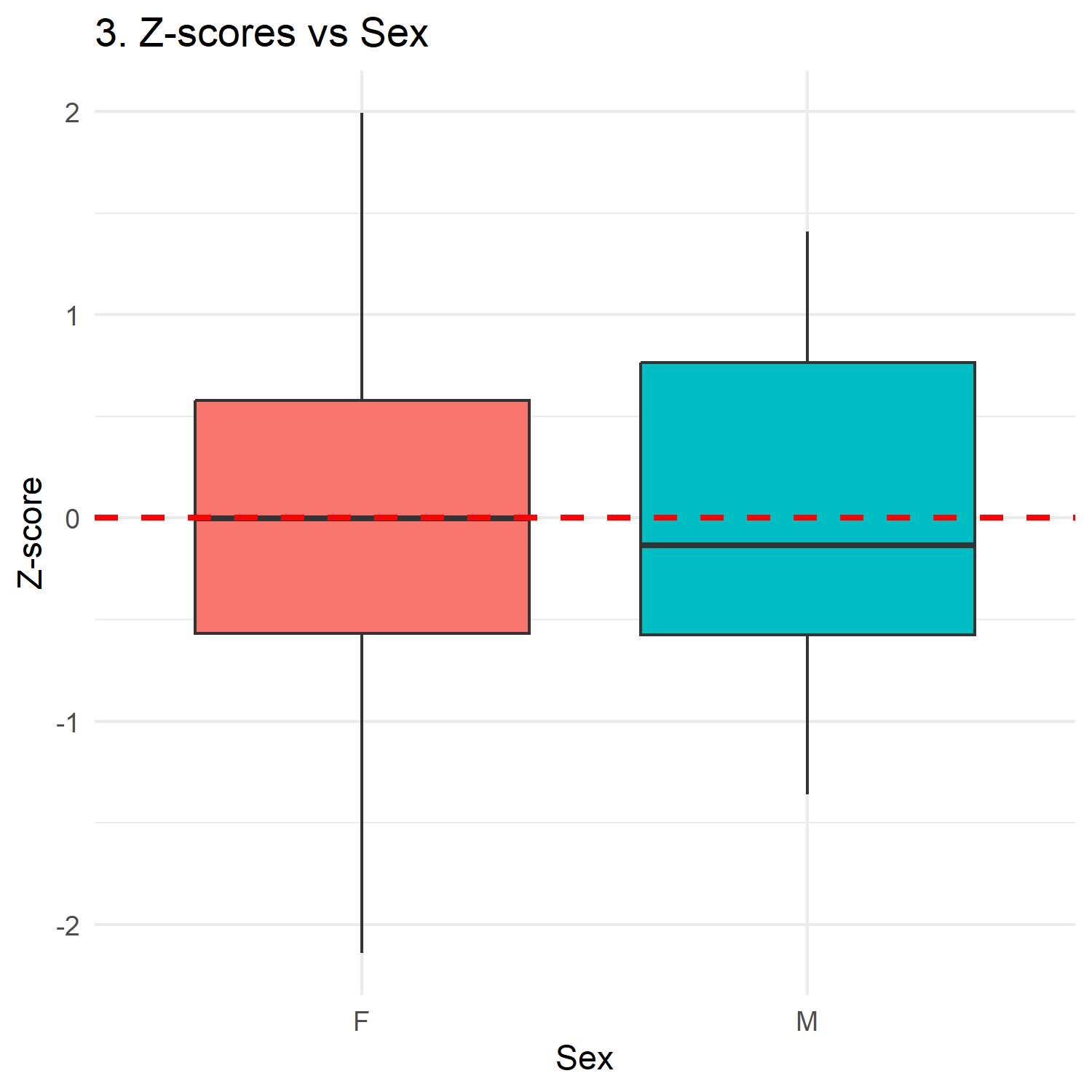
**

**A B**

Legend: (A)The plot assesses the removal of the Age effect by the GAMLSS model. The LOESS regression curve (red line) is centered closely to Z=0, confirming that the majority of the age-related effect was successfully accounted for. Minor residual curvature observed at the age extremes is attributed to reduced sample density in these ranges, while maintaining the model's stability. (B) The plot verifies that the systematic influence of Sex has been fully removed from the standardized values. Box plots for both Male (M) and Female (F) groups show medians centered on Z=0 (red dashed line), confirming that the Z-scores are independent of the Sex variable.

**s.Figure 3.** Diagnostic Plot for Standardized Z-scores (Z_NfL) vs Age (HC)

)


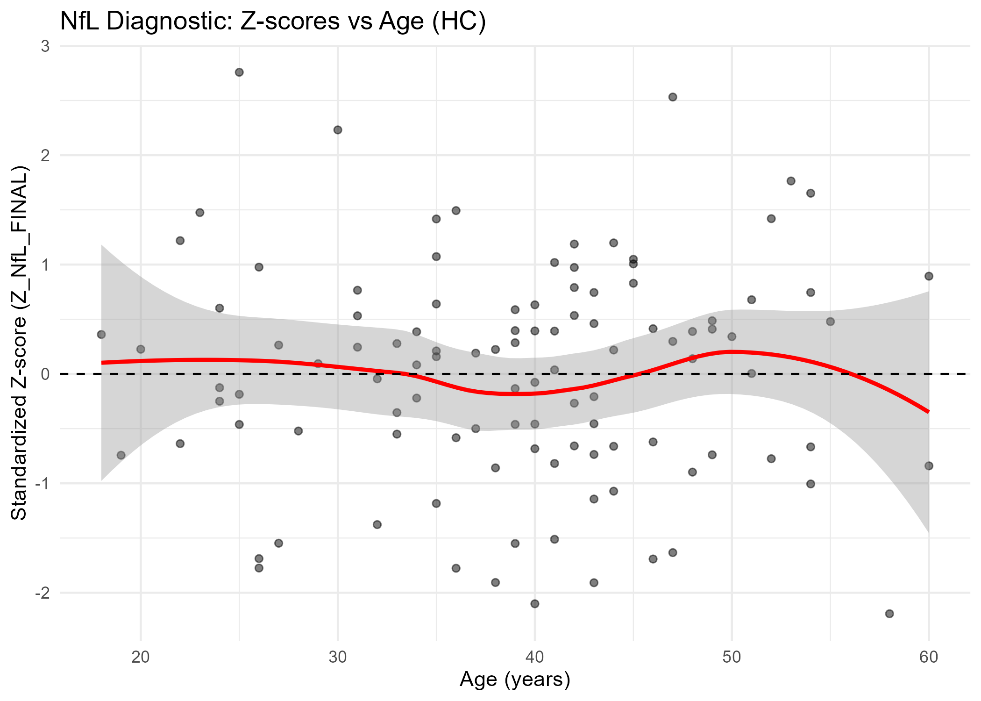

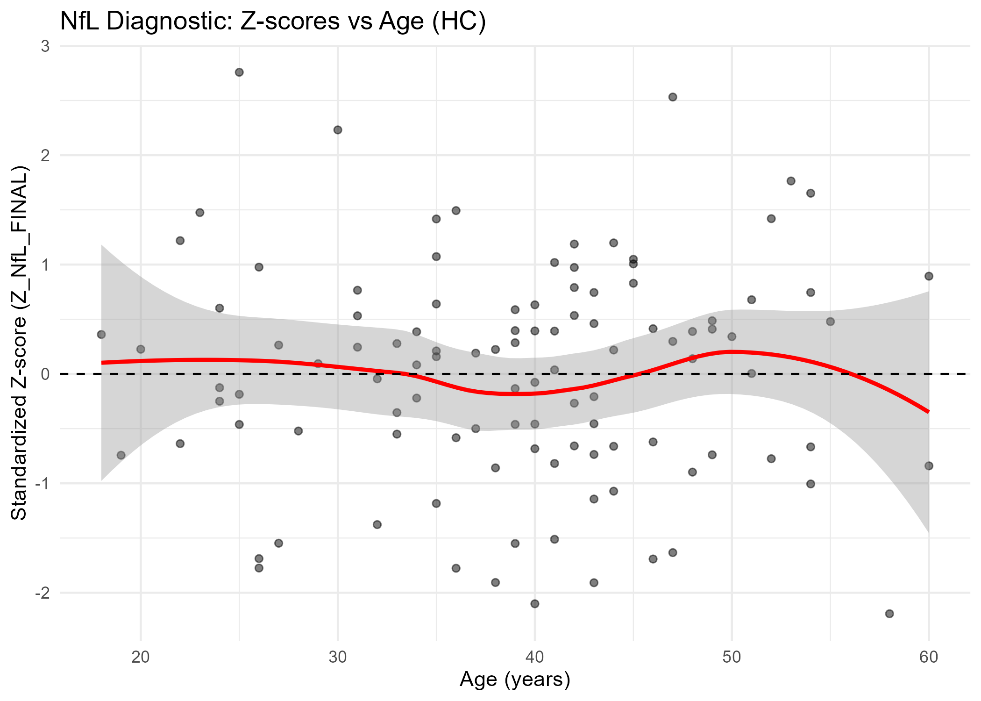


Legend: The plot assesses the removal of the Age effect by the GAMLSS model. The LOESS regression curve (red line) is centered closely to Z=0, confirming that the majority of the age-related effect was successfully accounted for. Minor residual curvature observed at the age extremes is attributed to reduced sample density in these ranges, while maintaining the model's stability.

**s.Figure 4.** Age-related sGFAP (pg/mL) Reference Centile Curves in Healthy Controls (HC)


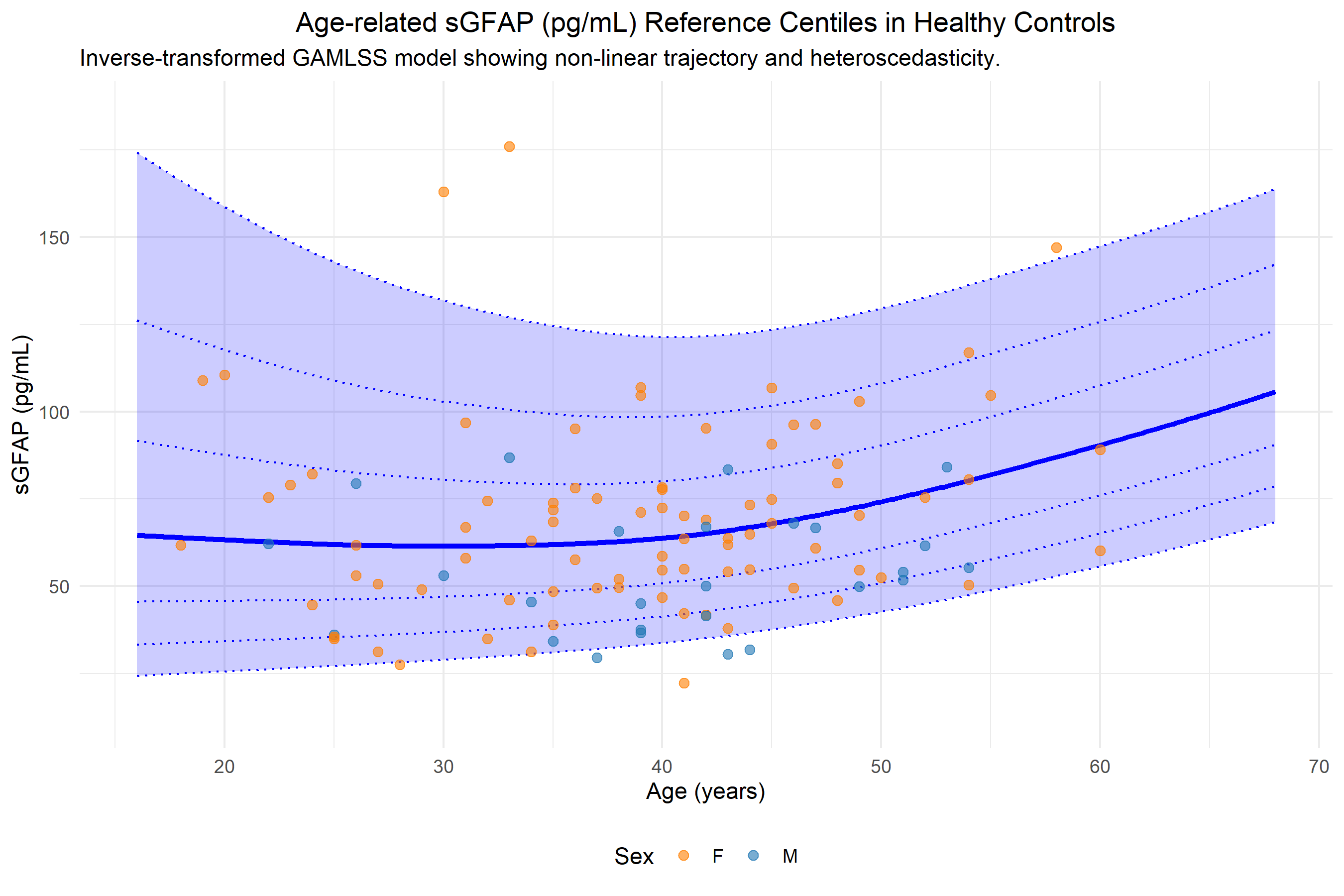


Legend: Graphical representation of the association between sGFAP and Age. The solid blue line represents the median (50^th percentile) of log-transformed sGFAP levels, back-transformed to the original scale (pg/mL). Dotted lines illustrate key centile curves (3^rd, 10^th, 25^th, $75^th, 90^th, and 97^th), with the shaded area covering the 94% reference range (3^rd to 97^th centile). Data points are individual HC observations, colored according to Sex. The figure shows the non-linear trajectory of sGFAP and the heteroscedasticity (age-dependent variance) successfully modeled by the GAMLSS framework.

**s.Figure 5.** Age-related sNfL (pg/mL) Reference Centile Curves in Healthy Controls (HC)

**
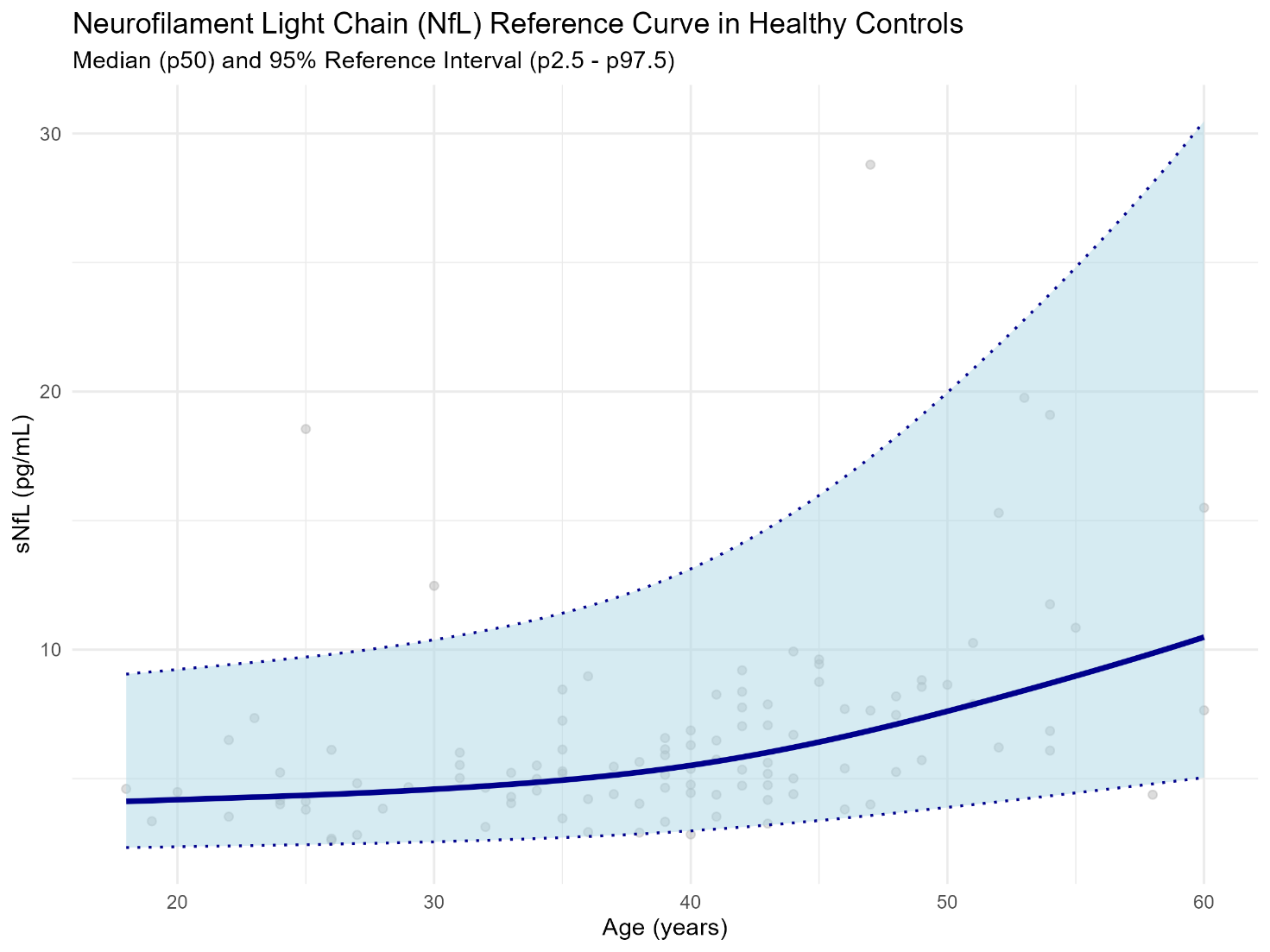
**

Legend: Graphical representation of the association between sNfL and Age. The solid blue line represents the median (50^th percentile) of log-transformed sNfL levels, back-transformed to the original scale (pg/mL). Dotted lines illustrate key centile curves (2.5^rd – 97.5^th), with the shaded area covering the 95% reference range. Data points are individual HC observations. The figure shows the non-linear trajectory of sNfL and the heteroscedasticity (age-dependent variance) successfully modeled by the GAMLSS framework.

**s.Figure 6.** External Validation and Agreement Analysis of Locally Derived NfL Z-scores vs Standardized Reference in HC and MS.

**
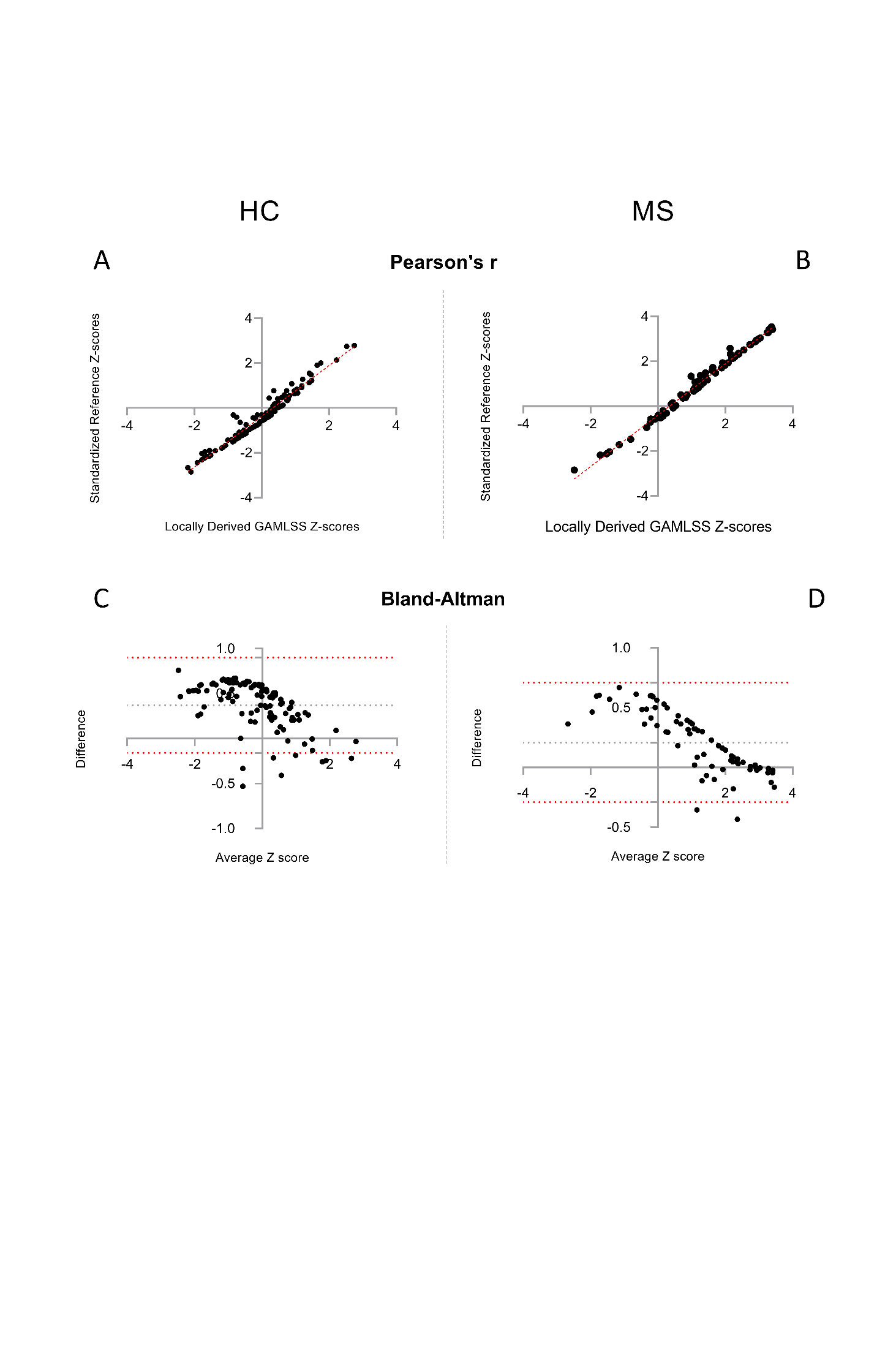
**

| Pearson | r | 95% CI | p-vlaue | R^2 |
| --- | --- | --- | --- | --- |
| A) HC | 0.978 | 0.969 / 0.980 | <0.0001 | 0.957 |
| B) MS | 0.994 | 0.991 / 0.996 | <0.0001 | 0.989 |
| **Bland-Altman** | **Bias** | **SD BIAS** | **95 % LOA** | |
| C) HC | 0.373 | 0.272 | -0.161 / 0.907 | |
| D) MS | 0.208 | 0.257 | -0.295 / 0.711 | |

Legend: Pearson Correlation Analysis. These scatter plots evaluate the linear relationship between the Locally Derived GAMLSS Z-scores (Model Z-scores X-axis) and the Standardized Reference Z-scores (Reference Z-scores, Y-axis) (16) for the Healthy Control (HC) cohort (A) and the Multiple Sclerosis (MS) cohort. In dotted red lines the linear regression line. Bland-Altman Agreement Analysis. Plots assess the level of agreement between the two Z-score methods for the HC cohort (C) and the MS cohort (D). In the Y-axis, the difference between the two methods (Local Z-score - Reference Z-score), vs the average Z-score (X-axis). Dotted grey lines the mean bias. Dotted red lines the 95% Limits of Agreement (LOA). The tight clustering around the bias line confirms the strong agreement.
